# Effects of intracranial artery stenosis of anterior circulation on cognition—A CT perfusion‐based study

**DOI:** 10.1002/brb3.3521

**Published:** 2024-09-05

**Authors:** Shanshan Yin, Ying Zhang, Baogen Du, Shanshan Cao, Kai Wang, Qiang Wei

**Affiliations:** ^1^ Department of Neurology The First Affiliated Hospital of Anhui Medical University Hefei Anhui China; ^2^ Anhui Province Key Laboratory of Cognition and Neuropsychiatric Disorders Hefei Anhui China; ^3^ Collaborative Innovation Center of Neuropsychiatric Disorders and Mental Health Hefei Anhui China; ^4^ Institute of Artificial Intelligence, Hefei Comprehensive National Science Center Hefei Anhui China; ^5^ The School of Mental Health and Psychological Sciences Anhui Medical University Hefei Anhui China

**Keywords:** benign oligemia, cerebral blood flow, cognitive impairment, CT perfusion, intracranial atherosclerotic stenosis, ischemic penumbra

## Abstract

**Background:**

Intracranial atherosclerotic stenosis (ICAS) is one of the most important independent risk factors for stroke that is closely related to the occurrence of cognitive impairment. The relationship between ICAS and vascular cognitive impairment (VCI) remains unclear. Cerebral hemodynamic changes are one of the main causes of cognitive impairment. Computed tomographic perfusion (CTP) imaging can quantitatively analyze cerebral blood perfusion and quantify cerebral hemodynamic changes. Previous research on the relationship between hypoperfusion induced by ICAS and cognitive impairment, as well as its underlying mechanisms, remains relatively insufficient. This study is dedicated to elucidating the characteristics and potential mechanisms of cognitive impairment in ICAS patients with abnormal perfusion, utilizing CTP imaging as our primary investigative tool.

**Methods:**

This study recruited 82 patients who suffer from non‐disabling ischemic stroke (IS group) and 28 healthy controls. All participants underwent comprehensive neuropsychological assessments both collectively and individually, in addition to CTP imaging. Within the patient group, we further categorized individuals into two subgroups: the ischemic penumbra group (IP, *N* = 28) and the benign oligemia group (BO, *N* = 54), based on CTP parameters—*T*_max_. The correlations between cognitive function and abnormal perfusion were explored.

**Results:**

The cognitive function, including the overall cognitive, memory, attention, executive functions, and language, was significantly impaired in the IS group compared with that in the control group. Further, there are statistical differences in the stroop color–word test‐dot (Stroop‐D) and Montreal Cognitive Assessment (MoCA) sub‐items (memory + language) between the BO and IP groups. In the BO group, the score of Stroop‐D is lower, and the MoCA sub‐items are higher than the IP group. There is no correlation between CTP parameters and cognitive function.

**Conclusion:**

Cognitive function is significantly impaired in patients with ICAS, which is related to cerebral perfusion. Executive, memory, and language function were better preserved in ICAS patients without IP. Hence, this study posits that managing hypoperfusion induced by ICAS may play a pivotal role in the development of VCI.

## Graphical abstract

The results elucidate that executive, memory, and language function were better in ICAS patients without ischemic penumbra compared with the ischemic penumbra group. “b” and “c” show that there is no significant difference in memory and language function (*p *= .054; *p *= .06) between the non‐ischemic penumbra group and the ischemic penumbra group. “a” and “d” show that there are statistical differences in Stroop‐D and MoCA sub‐items (memory + language) between the NIP and IP groups (*p *= .042; *p* = .046). Hence, this study suggests that controlling ICAS‐induced hypoperfusion may be involved in the occurrence of VCI.

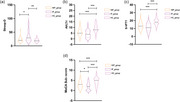

## 1 INTRODUCTION

Intracranial atherosclerotic stenosis (ICAS) is the most common cause of ischemic stroke (IS), especially in Asian countries, where it accounts for 33%–67% of strokes and transient ischemic attacks (TIAs) (Ritz et al., 2014; Shin et al., 2018). Studies have shown that symptomatic ICAS reports a higher prevalence (62%–69%) than asymptomatic ICAS (6%–13%) in Asia, much higher than in Europe and the United States (10%–16%). Despite optimal medical management of ICAS, the risk of recurrent IS and TIA remains high, occurring at a rate of up to 25% in the first year after an initial stroke (Wong & Li, 2003). The high incidence and high recurrence of symptomatic ICAS‐related IS pose a significant global burden (Gutierrez et al., 2022; Suri et al., 2016).

The most common sites of intra‐ and extracranial atheroscleroses are the basilar artery, internal carotid artery (ICA), middle cerebral artery (MCA), intracranial vertebral artery, posterior cerebral artery, and anterior cerebral artery (ACA). The anterior circulatory system comprises the ICA, MCA, ACA, and their related branches. This system supplies blood to brain regions crucial for cognitive function.

Severe stenosis or occlusion of blood vessels in the anterior circulatory system can lead not only to IS but also to poststroke cognitive impairment. This combination significantly reduces the quality of life for patients and their families (Qureshi & Caplan, 2014; Wang et al., 2019; Xie et al., 2021; Zhang et al., 2023).

Previous research has reported that symptomatic ICAS‐related IS often causes vascular cognitive impairment (VCI) except physical disability (Hilal et al., 2017; Iadecola et al., 2019; Qureshi & Caplan, 2014), which refers to the entire spectrum of vascular brain pathologies that contribute to any degree of cognitive impairment, ranging from subjective cognitive decline to dementia (Van Der Flier et al., 2018). Currently, studies have shown that patients with ICAS have cerebral hemodynamic damage, which is associated with cognitive impairment by causing cerebral hypoperfusion (Kim et al., 2012; Suri et al., 2018). The occurrence of ICAS has been attributed to vascular risk factors such as hypertension and diabetes (Gutierrez et al., 2022; Holmstedt et al., 2013)—the same risk factors that are also linked with VCI (Dearborn et al., 2017; Santisteban & Iadecola, 2018). Therefore, it is necessary to explore the connection between ICAS and cognitive impairment. Nonetheless, current research on the relationship between hypoperfusion caused by ICAS and cognitive impairment and its mechanism is relatively insufficient.

Cerebral blood flow (CBF) serves as a crucial indicator of the brain's blood supply. Computed tomographic perfusion (CTP) imaging can elucidate the extent of the irreversibly injured brain in the ischemic core, potentially salvageable but hypoperfusion ischemic penumbra (IP) and benign oligemia (BO). BO is defined as an ischemic area capable of spontaneous recovery, with CBF below the normal range but no risk of infarction. Among the CTP parameters, the time to maximum (*T*_max_) offers valuable insights into the assessment of the IP (Giammello et al., 2022; Lansberg et al., 2012; Li et al., 2013; Smith & Rowland Hill, 2018).

In this cross‐sectional study, we quantified alterations in cerebral perfusion to investigate the relationship between hypoperfusion induced by ICAS and cognitive impairment. We employed cognitive screening and conducted CTP scans in patients who had experienced non‐disabling ISs related to ICAS.

## 2 MATERIALS AND METHODS

### 2.1 Participants

In this study, we enrolled 82 patients (Ischemic Stroke group; IS group) with unilateral (BO Group: 54; IP Group: 28) severe (>70%) atherosclerotic stenosis or occlusion of the intracranial ICA (C6 or C7 segment) or MCA (M1 segment) and 28 healthy controls. Patients with a time to maximum of no more than 6 s (*T*_max_ < 6 s) were defined as the BO group, and patients with a time to maximum more than 6 s (*T*_max_ > 6 s) were defined as IP group, respectively (Giammello et al., 2022; Lansberg et al., 2012).

ICAS encompasses a spectrum from wall thickening and gradual plaque growth to severe lumen stenosis. It can be diagnosed using digital subtraction angiography (DSA), the traditional criterion for assessing cerebral vasculature. Magnetic resonance angiography (MRA) and computed tomography angiography (CTA) results suggest the possibility of intracranial vascular stenosis in patients, and after communication with patients and their families, DSA examinations have been further confirmed (Chen et al., 2024; Nguyen‐Huynh et al., 2008).

Inclusion criteria included that patients (1) were between 18 and 70 years of age, (2) had severe (>70%) stenosis or occlusion of the intracranial segment of the ICA (C6 or C7 segment) or MCA (M1 segment) diagnosed by CTA, MRA, or DSA, (3) were nondisabled, with a modified Rankin Scale (mRS) of <3 or National Institute of Health Stroke Scale (NIHSS) score of <4, (4) had no new ischemic cerebrovascular events, such as cerebral infarction or TIA, within the past 14 days, and (5) were right‐handed. Patients were excluded if they (1) had other diseases and factors affecting cognition, such as intracranial hemorrhage, brain tumor, normal intracranial pressure hydrocephalus, mental disease and drug abuse caused by Alzheimer's disease, Parkinson's disease, brain trauma, and others, (2) had severe visual, hearing, language and physical dysfunction, and unable complete the corresponding test scale, (3)were illiteracy, and declined neuropsychological evaluation, or a non‐coordinated evaluation process, (4) had CTP contraindications or factors affecting imaging quality, (5) had massive cerebral infarction or cortical infarction, (6) had non‐atherosclerotic stenosis (including but not limited to moyamoya disease and vasculitis, and (7) a history of head and neck stenting, balloon dilatation, internal carotid endarterectomy, aneurysm embolization, arteriovenous malformation embolization, or head‐and‐neck open surgery. The patients were outpatients in the Department of Neurology or inpatients in the Department of Neurology Ward of the First Affiliated Hospital of Anhui Medical University from 2018 to 2023. The participants in the control group were healthy individuals whose age, sex, and years of education matched the patient groups.

### 2.2 CTP data acquisition

Scanning with GE Revolution 256‐slice CT scanner, iodinated contrast (350 mgL/mL; 100 mL) was injected at 4 mL/s (total acquisition time, 51 s). Scanning parameters are as follows: 80 kV, 100 mA, field of view = 100 mm × 100 mm, window width = 300, window length = 30, layer thickness = 5 mm. Contrast tracking triggers thin‐layer reconstruction of the post‐scan raw data rows and transfer to a GE AW4.7 workstation for processing.

### 2.3 Data preprocessing

CTP imaging has evolved, and fully automated, standardized volumetric processing can now be rapidly performed with RAPID software (Bivard et al., 2011, 2013; Campbell et al., 2015). The CTP data is seamlessly transmitted to a networked computer equipped with the automated RAPID software which performs motion correction, generates maps and lesion segmentation, and then sends processed images to the hospital's Picture Archiving and Communication System. Furthermore, the RAPID system computes quantitative perfusion maps using deconvolution of tissue and arterial signals. In RAPID, the brain regions with altered perfusion were identified on the *T*_max_ map (Campbell et al., 2015; Straka et al., 2010).

### 2.4 T_max_

The *T*_max_ parameter, one of the parameters of CTP, is an estimate of the time delay in blood delivery between the main supplying artery and tissue at a given spatial location, independent of shape. Brain tissue at risk for infarction—IP was distinguished from minimally hypoperfusion tissue if the *T*_max_ delay was more than 6 s (Giammello et al., 2022; Lansberg et al., 2012).

### 2.5 CBV index

Cerebral blood volume (CBV) index means a relative CBV of *T*_max_ > 6 s, which is associated with collateral status and infarct growth and would be useful as a parameter to predict underlying ICAS (Imaoka et al., 2023; Karamchandani et al., 2022; Potreck et al., 2022).

### 2.6 Research design and process

In this study, patients were divided into the BO group and the IP group by *T*_max_. Trained neurologists and neurology postgraduates conducted the screening and examination of subjects. The participants were patients with non‐disabling intracranial artery stenosis or occlusion. Non‐disabling was defined as a baseline NIHSS score of ≤5, and each baseline NIHSS score item (items 1a–1c were 0) was scored 0 or 1 (Wang et al., 2019), or a mRS score of 0–2 (Roussopoulou et al., 2019). In addition, we collected CT perfusion data for all participants and assessed their cognitive function. We completed the statistical analysis after preprocessing the relevant data.

### 2.7 Cognitive assessments

The participants were required to complete all cognitive assessments, including global and individual cognitive assessments. The Montreal Cognitive Assessment (MoCA) was used to assess the global cognitive function, and the Chinese version of the auditory verbal learning test (AVLT) (Lees et al., 2014; Schoenberg et al., 2006), comprising immediate, delayed, and recognition memory functions, was used to assess memory function. The digital span test (forward/backward) was used to assess the participant's attention function, and the semantic verbal fluency test (S‐VFT: S‐VFT1, vegetables and fruits; S‐VFT2, animals) was used to assess the fluency for frontal lobe function and language function. The stroop color–word test (SCWT‐dot, word, and colored word) was used to assess the executive function, and the Color Trail Test A/B was used to assess the executive and attention functions, respectively.

### 2.8 Statistical analysis

The differences in age, years of education, and cognitive assessment results were assessed with one‐way analysis of variance (ANOVA) (normally distributed data) or the rank sum test (non‐normally distributed data) and using the Welch ANOVA to assess the data that failed in homogeneity‐of‐variance. Differences in sex distribution, diabetes, and hyperlipidemia between the healthy control group and patient groups were assessed with the Pearson χ2 test using the Statistical Package for the Social Sciences 26 (SPSS). The extracted values were introduced into SPSS 26, and a one‐way ANOVA analyzed the differences between the groups. The least significant difference (LSD, when the variance was homogeneous) and Tamhane's T2 (when the variance was uneven) were used in one‐way ANOVA for post hoc analysis, while the Bonferroni correction was used in the chi‐square test and rank sum test (significant for *p* < .05). Meanwhile, vascular risk factors were used as covariables for partial correlation analysis to determine the correlation between the CTP parameters and cognitive functional outcomes.

## 3 RESULTS

### 3.1 Demographics and clinical characteristics

There were no significant differences in age, years of education, or sex distribution between the groups. The incidence of hypertension and hyperlipidemia was lower in the control group than in the IP group, but there were no significant differences in the incidence of diabetes or smoking history between the groups. The demographic and clinical characteristics of the participants are shown in Table 1. The kinds and duration of medications that patients took were recorded, as detailed in Table S2.

**TABLE 1:** Demographic data and clinical characteristics of the study participants.

	BO (*n* = 54)	IP (*n* = 28)	HC (*n* = 28)	*F*/*x* ^2^	*p*‐Value
Age, years	54.52 ± 8.07	55.89 ± 11.54	58.57 ± 6.74	2.835	.067
Educational, years	6.52 ± 3.57	6.75 ± 4.02	8.59 ± 3.07	3.195	.051
Gender (M/F)	40/14	23/5	17/11	3.137	.208
Hypertension	33/15	19/4^a^	11/16	10.299	.006^*^
Diabetes	10/38	7/16	2/25	4.340	.114
Hyperlipidemia	23/19	16/5^a^	9/17	8.104	.017^*^
Smoking history	20/27	7/16	14/14	2.415	.660

*Note*: Data are presented as mean ± standard deviation.

Abbreviations: BO, benign oligemia group; HC, healthy control group; IP, ischemic penumbra group.

^a^ Control group versus IP group (*p *< .05).

* Significant at 0.05 level (two‐tailed).

### 3.2 Cognitive assessments

**FIGURE 1:**
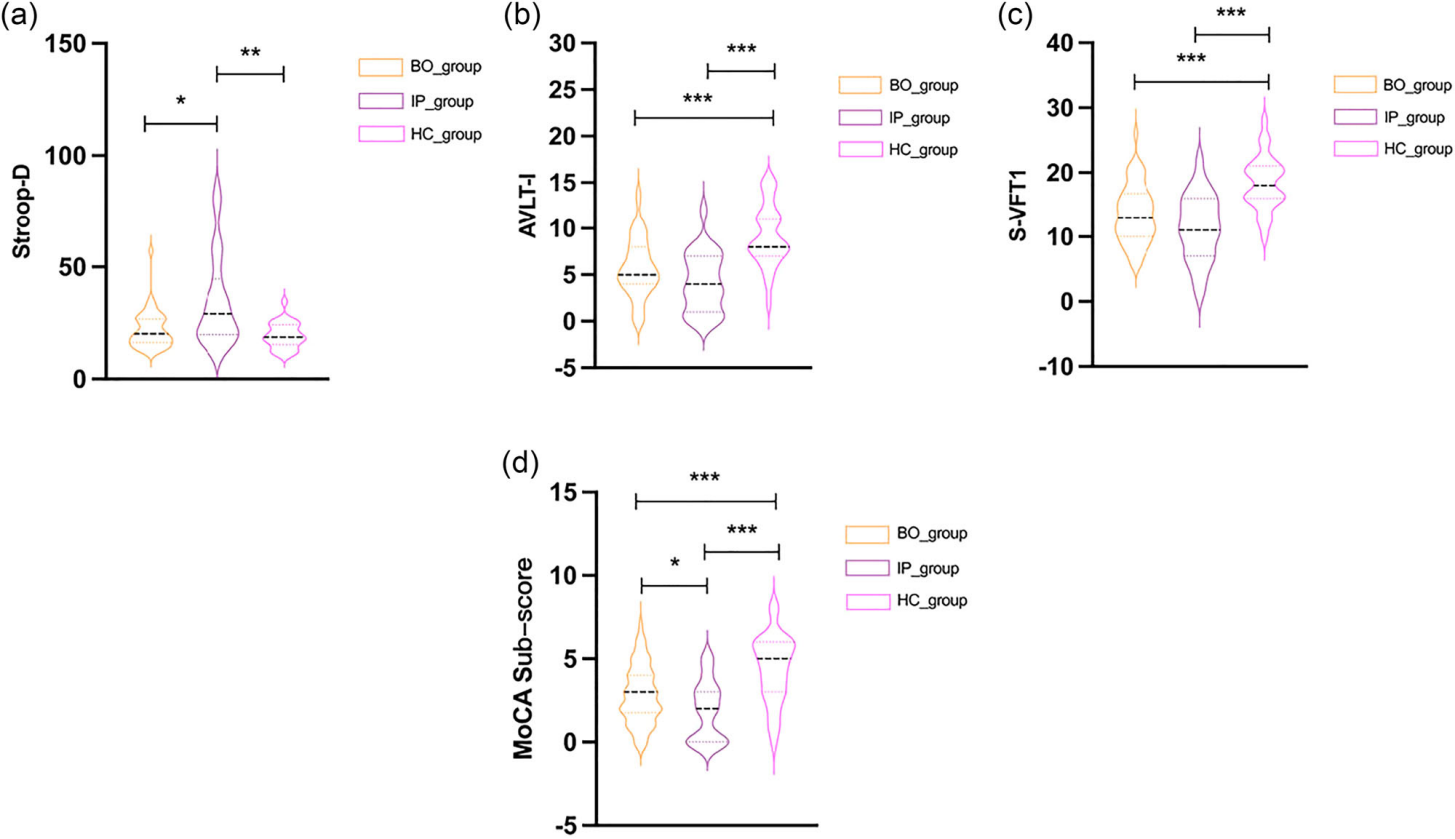
Cognitive function assessments (a–d) in patients with intracranial atherosclerotic stenosis (ICAS). HC, healthy control group; BO, benign oligemia group; IP, ischemic penumbra group. *Significant at 0.05 level (two‐tailed), **significant at 0.01 level (two‐tailed), ***significant at 0.001 level (two‐tailed).

The cognitive ability, including the overall cognitive, memory, attention, executive, and language functions, was significantly impaired in the patient groups compared with that in the control group. There is no significant difference in memory and language function (*p *= .054; *p* = .06) between BO and IP groups (shown in Table 2 and Figure 1b,c). There are statistical differences in Stroop‐D and MoCA sub‐items (memory + language) between the BO and IP groups (*p *= .042; *p* = .046). The results are shown in Figure 1a,d.

**TABLE 2:** Cognitive function assessments and computed tomographic perfusion (CTP) parameters of the study participants.

	BO (*n* = 53)	IP (*n* = 28)	HC (*n* = 28)	*F*/*F*′	Hc	*p*‐Value
MoCA MoCA Sub‐items	18.96 ± 4.30^a^	17.50 ± 4.81^b^	23.52 ± 2.42	26.92		.000^***^
	2.81 ± 1.73^c^	1.86 ± 1.78	4.39 ± 2.02		21.06	.000^***^
**Memory function**						
AVLT‐I	5.69 ± 3.17^a^	4.16 ± 3.21^b^	9.18 ± 3.36	17.54		.000^***^
AVLT‐D	5.06 ± 3.2^a^	3.69 ± 2.90^b^	9.04 ± 3.54	20.98		.000^***^
AVLT‐R	11.31 ± 3.09^a^	11.00 ± 3.28^b^	13.56 ± 1.37		14.84	.001^***^
**Attention function**						
DST‐F	7.70 ± 1.31	7.82 ± 1.66	7.46 ± 1.64			
DST‐B	3.62 ± 1.15^a^	3.93 ± 1.74	4.36 ± 1.19		6.92	.031^*^
**Executive function**						
Stroop‐D	22.09 ± 8.58^c^	34.27 ± 20.39^b^	19.36 ± 5.75		11.18	.004^**^
Stroop‐W	28.43 ± 11.31	40.43 ± 24.05^b^	23.18 ± 6.96		9.93	.007^**^
Stroop‐CW	43.19 ± 19.05	55.65 ± 29.60^b^	36.76 ± 10.15		6.18	.046*
CTT A	91.31 ± 37.53^a^	128.55 ± 65.84^b^	64.62 ± 24.10		18.96	.000^***^
CTT B	160.00 ± 62.63^a^	204.47 ± 106.1^b^	123.21 ± 58.89	5.63		.007^**^
**Langue function**						
S‐VFT1	13.52 ± 4.46^a^	11.44 ± 5.16^b^	18.35 ± 4.23	16.01		.000^***^
S‐VFT2	13.25 ± 4.83	11.78 ± 5.09^b^	15.07 ± 6.27		8.91	.012*
**CTP parameters**						
V‐*T*_max_4s	36.55 ± 42.28	238.07 ± 132.34	–			
V‐*T*_max_6s	–	74.14 ± 77.60	–			
V‐*T*_max_8s	–	23.75 ± 42.66	–			
V‐*T*_max_10s	–	8.18 ± 23.65	–			
Mismatch	–	72.25 ± 78.38	–			
CBV index	–	0.7429 ± 0.16	–			
NIHSS	0.44 ± 0.84	0.50 ± 1.14	–		460.5	.546

*Note*: Data are presented as mean ± standard deviation. Mismatch, the ratio of ischemic tissue volume to core infarction volume; CBV (cerebral blood volume) index, the ratio of average CBV of brain tissue volume with *T*_max_ > 6 s to average CBV of healthy brain tissue.

Abbreviations: AVLT‐I/D/R, Chinese Auditory Learning Test, immediate/delayed/recognition memory functions; BO, benign oligemia group; CTT, Color Trail Test; DST‐F/B, digital span test forward/backward; HC, healthy control group; IP, ischemic penumbra group; MoCA sub‐items, the sum of memory and language function scores; MoCA, Montreal Cognitive Assessment; NIHSS, National Institute of Health Stroke Scale; S‐VFT, semantic verbal fluency test; V‐*T*_max_, the volume of time to maximum.

^a^ control group versus BO group (*p *< .05).

^b^ control group versus IP group (*p *< .05).

* Significant at 0.05 level (two‐tailed).

** Significant at 0.01 level (two‐tailed).

*** Significant at 0.001 level (two‐tailed).

### 3.3 CTP data analysis

There exists no correlation between CTP parameters and cognitive function. The results are shown in Table S1.

## 4 DISCUSSION

In this study, we used CTP to clarify the cerebral perfusion changes in patients with ICAS‐related non‐disabling IS by volume of *T*_max_ and further explore the relationship between hypoperfusion and cognitive impairment caused by ICAS. To investigate the cognitive impairment caused by hypoperfusion, the patients were separated into BO and IP groups by the volume of *T*_max_. Cognitive function is significantly impaired in patients with the IS group, which is related to cerebral perfusion. There is a statistical difference in the stroop color–word test‐dot (Stroop‐D). Although there is no significant difference in language and memory function between the BO and IP groups (.05 < *p* < .1), the MoCA sub‐items are significantly different between the BO and IP groups (*p *= .046), which means the cognitive functions in the MoCA sub‐items without IP are better preserved (Coleman et al., 2017). Current studies have shown that the cognitive impairment of ICAS‐related stroke is mainly manifested in executive function, in addition to language and memory (Frisoni et al., 2002; Sun et al., 2016).

Vascular risk factors, such as hypertension, diabetes, and smoking, can exacerbate the pathological process of ICAS (Iadecola et al., 2019), which are also linked with AD and VCI. Autopsy studies of cerebral arteries have shown that any degree of endometrial thickening causes the lumen to shrink proportionally (Pasterkamp et al., 1997). Among patients with symptomatic and asymptomatic ICAS, uncontrolled vascular risk factors are associated with the severity of stenosis and an increased risk of stroke (Gutierrez et al., 2022). In this study, we collected vascular risk factors, such as hypertension, diabetes, hyperlipidemia, and smoking history, which were included in the analysis as covariates (Paulson & Vigdis, 2020). The results clarify that the BO group exhibits superior retention of executive function compared to the IP group. This finding has valuable implications for guiding clinical practice.

Previous studies have indicated that ICAS is an important risk factor for cognitive impairment (Van Der Flier et al., 2018). The potential mechanisms of cognitive dysfunction include cerebral ischemic injury and cerebral hypoperfusion (Deng et al., 2018; Oudeman et al., 2018).

Studies have pointed out that hypoperfusion will produce a state of hypoxia, so the resulting energy imbalance will cause spontaneous and continuous depolarization and overstimulation of neurons, through compensation, further destroy the oxygen balance, leading to the dominance of pro‐oxidant species, and finally produce oxidative stress environment, which is one of the important factors related to cognitive decline (Arundine & Tymianski, 2003; Tanović & Alfaro, 2006). Additionally, reduced CBF can cause cell damage, activate glial cells, induce apoptosis in neighboring cells, and ultimately compromise the integrity of the blood‐brain barrier. Although the relationship between blood–brain barrier injury and cognitive decline is well established, the exact neuropathological changes involved remain unclear (Rajeev et al., 2023; Xiong et al., 2004).

Various symptoms of cognitive impairment may limit the ability of daily living, such as memory loss, dysfunctions of verbal communication, learning disorders, concentration problems, and intellectual disabilities. The progressive cognitive decline in patients with ICAS should be carefully detected, as it may impose other burdens on the patient's life besides stroke. One study pointed out that decreased CBF can destroy neuronal microenvironment homeostasis and aggravate neurological dysfunction in Alzheimer's disease, which can cause cognitive dysfunction (Kisler et al., 2017; Ogoh, 2017). These findings provide evidence that the decline of cognitive function in patients with ICAS may be affected by hypoperfusion caused by ICAS. Consistent with the above, our study found that ICAS‐induced IP is associated with executive, language, and memory function, and ICAS‐induced hemodynamic disturbance is an important factor in the occurrence of VCI.

Despite the careful participant enrollment and data analysis, this study has some limitations. First is the relatively small sample size, which may affect the results to a certain extent. Second, is its cross‐sectional nature, which makes it difficult to obtain a strong causal relationship from the study itself. Cognitive function is determined not only by IP but also by other factors. Third, the locations of atherosclerotic stenosis and IP were different in ICAS patients. Fourth, the difference between the anterior and posterior cycles and the lateralization of the brain were not explored. Fifth, the infarct sites of the patients included in this study are not completely consistent, which may affect the result of the study. Therefore, to reduce the impact, patients with non‐disabling IS were selected for this study. However, it is still a limitation of the study. In addition, this study did not include the stenosis or occlusion of the ACA, which may affect the result. We believe that, compared with ACA, the blood‐supplying brain areas of MCA and ICA overlaps more (Everts et al., 2014; Wang et al., 2016). To achieve better homogeneity of inclusion patients, the inclusion vessels were limited to MCA and ICA. Moreover, this study is a cross‐sectional study. We selected ICA and MCA segments because we want to further explore the effects of PTA and PTS on cognitive function in the future. However, ACA has thinner blood vessels, and the risk of PTS is higher. In general, this is still the limitation of this study. Given the above limitations, a more accurate cognitive assessment and a larger sample size should be conducted in the future.

## 5 CONCLUSION

Cognitive function is impaired in patients with ICAS related to cerebral perfusion. Executive, memory, and language function were better in ICAS patients without IP compared with the IP group. Hence, this study suggests that controlling ICAS‐induced hypoperfusion may be involved in the occurrence of VCI.

## AUTHOR CONTRIBUTIONS

**Shanshan Yin**: Conceptualization; writing—original draft; formal analysis. **Ying Zhang**: Writing—original draft; formal analysis. **Baogen Du**: Data curation. **Shanshan Cao**: Writing—review and editing; methodology. **Kai Wang**: Conceptualization; writing—review and editing. **Qiang Wei**: Methodology; writing—review and editing.

## CONFLICT OF INTEREST STATEMENT

The authors declare that the research was conducted in the absence of any commercial or financial relationships that could be construed as a potential conflict of interest.

### PEER REVIEW

The peer review history for this article is available at https://publons.com/publon/10.1002/brb3.3521.

## INFORMED CONSENT

Informed consent was obtained from all individual participants included in the study.

## Supporting information


**Table S1** Correlation analysis between CBV Index and cognitive function in the ischemic penumbra Group.
**Table S2** The kinds and duration of medication of the patients.

## Data Availability

All data generated or analyzed during this study are included in this article. Further inquiries can be directed to the corresponding author.
