# Supplementary material for: Effects of intracranial artery stenosis of anterior circulation on cognition—A CT perfusion‐based study
Source: Brain Behav. 2024 Sep 5;14(9):e3521. doi: 10.1002/brb3.3521 (PMC11376367; doi:10.1002/brb3.3521)
Supplement: Supplementary file 1 — Table S1 Correlation analysis between CBV Index and cognitive function in the ischemic penumbra Group. Table S2 The kinds and duration of medication of the patients. [file BRB3-14-e3521-s001.docx]

**Supplemental Table 1**. Correlation analysis between CBV Index and cognitive function in the Ischemic Penumbra Group.

|  | r-value | *p-*value | r-value | *p-*value | |
| --- | --- | --- | --- | --- | --- |
| MoCA  MoCA  Sub-items | -0.232 | 0.23^a^ | -0.583 | 0.10^b^ | |
|  | -0.137 | 0.51^a^ | -0.524 | 0.15^b^ | |
| **Memory**  **function** |  |  |  |  | |
| AVLT-I | -0.022 | 0.92^a^ | -0.602 | 0.09^b^ | |
| AVLT-D | 0.181 | 0.38^a^ | -0.264 | 0.49^b^ | |
| AVLT-R | -0.103 | 0.63^a^ | -0.655 | 0.06^b^ | |
| **Attention**  **function** |  |  |  |  | |
| DST-F | -0.332 | 0.09^a^ | -0.482 | 0.19^b^ | |
| DST-B | -0.333 | 0.08^a^ | -0.510 | 0.16^b^ | |
| **Executive**  **function** |  |  |  |  | |
| Stroop-D | 0.187 | 0.42^a^ | 0.224 | 0.56^b^ | |
| Stroop-W | 0.163 | 0.51^a^ | 0.341 | 0.37^b^ | |
| Stroop-CW | 0.207 | 0.40^a^ | 0.409 | 0.27^b^ | |
| CTT A | -0.084 | 0.72^a^ | 0.324 | 0.40^b^ | |
| CTT B | 0.044 | 0.86^a^ | 0.397 | 0.29^b^ |  |
| **Langue**  **function** |  |  |  |  | |
| S-VFT1 | -0.217 | 0.28^a^ | -0.293 | 0.44^b^ | |
| S-VFT2 | -0.190 | 0.34^a^ | -0.384 | 0.31^b^ | |

^a^, the simple correlation analysis method did not adjust the *p*-value obtained by vascular risk factors; ^b^, partial correlation analysis method, *p*-value after adjusting vascular risk factors (hypertension, diabetes, hyperlipidemia, etc.); The MoCA sub-item is the sum of memory and language function scores.

**Supplemental Table 2.** The kinds and duration of medication of the patients

|  | Hypertension | | Diabetes | | Hyperlipidemia | | Antiplatelet therapy | |
| --- | --- | --- | --- | --- | --- | --- | --- | --- |
|  | Kind | Duration | Kind | Duration | Kind | Duration | Kind | Duration |
| PT1-1 | compound reserpine tablets | >1825 | -- |  | Atorvastatin Calcium | 20 | DA | 20 |
| PT1-2 | Extended Release Nifedipine Tablets | 737 | Metformin，Glibenclamide，Glimepiride | 737；730；7 | -- |  | DA | 15 |
| PT1-3 | Missing |  | Missing |  | Missing |  |  |  |
| PT1-4 | Missing |  | Missing |  | Missing |  |  |  |
| PT1-5 | Indapamide；amlodipine | 90；24 | -- |  | -- |  | SA +Cilostazol | 24 |
| PT1-6 | Missing |  | Missing |  | Missing |  |  |  |
| PT1-7 | -- |  | -- |  | Atorvastatin Calcium | 32 | DA | 32 |
| PT1-8 | Indapamide | 7 | -- |  | 无 |  | DA | 32 |
| PT1-9 | -- |  | -- |  | Atorvastatin Calcium | 15 | DA | 15 |
| PT1-10 | -- |  | -- |  | 无 |  | DA | 18 |
| PT1-11 | -- |  | Metformin； Repaglinide； Rosiglitazone | 24 | Atorvastatin Calcium | 24 | DA | 24 |
| PT1-12 | -- |  | -- |  | -- |  | DA | 17 |
| PT1-13 | / | >1825 | -- |  | -- |  | DA | 15 |
| PT1-14 | -- |  | -- |  | Atorvastatin Calcium | 16 | SA + Cilostazol | 16 |
| PT1-15 | Missing |  | Missing |  | Missing |  |  |  |
| PT1-16 | amlodipine | 6 | -- |  | Atorvastatin Calcium | 16 | SA | 16 |
| PT1-17 | Irbesartan；Extended Release Nifedipine Tablets | 12；12 | -- |  | Atorvastatin Calcium | 18 | DA | 18 |
| PT1-18 | Extended Release Nifedipine Tablets | 9 | -- |  | Atorvastatin Calcium | 15 | DA | 15 |
| PT1-19 | Valsartan；amlodipine | 10 | -- |  | / |  | DA | 20 |
| PT1-20 | Amlodipine Besylate and Benazepril Hydrochloride Tablets; Irbesartan and Hydrochlorothiazide Tablets | 50；7 | -- |  | -- |  | DA | 20 |
| PT1-21 | / |  | -- |  | Atorvastatin Calcium | 51 | DA | 51 |
| PT1-22 | -- |  | Metformin；Acarbose | >1825；4 | / |  | DA | 15 |
| PT1-23 | / |  | -- |  | Rosuvastatin Calcium | 21 | DA | 21 |
| PT1-24 | amlodipine | 370 | -- |  | Atorvastatin Calcium | 85 | DA | 85 |
| PT1-25 | -- |  | -- |  | -- |  | DA | 17 |
| PT1-26 | Valsartan；Levamlodipine | >1825 | / |  | / |  | DA | 15 |
| PT1-27 | amlodipine | 5 | No medication |  | Rosuvastatin Calcium | 75 | DA | 75 |
| PT1-28 | -- |  | -- |  | / |  | DA | 15 |
| PT1-29 | -- |  | -- |  | / |  | DA | 17 |
| PT1-30 | -- |  | -- |  | -- |  | DA | 18 |
| PT1-31 | Extended Release Nifedipine Tablets | >1825 | -- |  | Atorvastatin Calcium | 83 | DA | 83 |
| PT1-32 | Valsartan； Felodipine | 1101 | Glimepiride； Acarbose | 1101 | Rosuvastatin Calcium；Atorvastatin Calcium | 77 | SA | 77 |
| PT1-33 | Extended Release Nifedipine Tablets | 3 | GlipizideTablets ；Metformin | 733 | Atorvastatin Calcium | 15 | DA | 15 |
| PT1-34 | -- |  | -- |  | -- |  | DA | 15 |
| PT1-35 | Missing |  | Missing |  | Atorvastatin Calcium | 25 | DA | 25 |
| PT1-36 | Metoprolol；Extended Release Nifedipine Tablets | 10;10 | Acarbose | 10 | -- |  | DA | 17 |
| PT1-37 | -- |  | -- |  | Atorvastatin Calcium | 15 | DA | 15 |
| PT1-38 | No medication |  | -- |  | Atorvastatin Calcium | 29 | DA | 29 |
| PT1-39 | -- |  | -- |  | Atorvastatin Calcium | 18 | DA | 18 |
| PT1-40 | -- |  | -- |  | Atorvastatin Calcium | 15 | DA | 15 |
| PT1-41 | Amlodipine Besylate and Benazepril Hydrochloride Tablets | >1825 | -- |  | -- |  | DA | 16 |
| PT1-42 | Irbesartan；Metoprolol ；Cilnidipine | >1825 | -- |  | -- |  | DA | 26 |
| PT1-43 | Extended Release Nifedipine Tablets；Candesartan ；amlodipine | 1095；1095；15 | -- |  | -- |  | DA | 15 |
| PT1-44 | Extended Release Nifedipine Tablets；Captopril | >1825 | -- |  | -- |  | DA | 16 |
| PT1-45 | Irbesartan ；Extended Release Nifedipine Tablets；amlodipine | >1825；>1825；3 | -- |  | Atorvastatin Calcium | 15 | DA | 15 |
| PT1-46 | -- |  | -- |  | / |  | DA | 17 |
| PT1-47 | Amlodipine Besylate | >1825 | -- |  | -- |  | DA | 42 |
| PT1-48 | / |  | Metformin ； Acarbose | 15 | / |  | DA | 22 |
| PT1-49 | Irbesartan ；Extended Release Nifedipine Tablets | 4 | No medication |  | -- |  | DA | 85 |
| PT1-50 | Felodipine ； Enalapril Maleate | >1825 | -- |  | Atorvastatin Calcium | 15 | DA | 15 |
| PT1-51 | Valsartan； amlodipine | 732 | -- |  | -- |  | DA | 17 |
| PT1-52 | Felodipine | >1825 | -- |  | Atorvastatin Calcium | 43 | DA | 43 |
| PT1-53 | / |  | -- |  | -- |  | DA | 22 |
| PT1-54 | amlodipine | 15 | -- |  | -- |  | DA | 20 |
| PT2-1 | Irbesartan | >1825 | -- |  | Atorvastatin Calcium | 88 | DA | 88 |
| PT2-2 | -- |  | -- |  | -- |  | SA | 18 |
| PT2-3 | Extended Release Nifedipine Tablets | >1825 | Metformin | >1825 | Atorvastatin Calcium | 16 | DA | 16 |
| PT2-4 | Missing |  | Missing |  | Missing |  |  |  |
| PT2-5 | Amlodipine Besylate | 124 | -- |  | Atorvastatin Calcium | 79 | DA | 79 |
| PT2-6 | No medication |  | -- |  | Atorvastatin Calcium | 19 | DA | 19 |
| PT2-7 | Felodipine 、Candesartan cilexetil | 150 | -- |  | -- |  | DA | 18 |
| PT2-8 | Amlodipine Besylate;Levamlodipine | 365；12 | Metformin；Acarbose | 365；12 | -- |  | DA | 18 |
| PT2-9 | Levamlodipine；amlodipine | 15；14 | Acarbose；Gliclazide；Metformin | 19 | / |  | DA | 29 |
| PT2-10 | -- |  | -- |  | Atorvastatin Calcium | 26 | DA +Cilostazol | 26 |
| PT2-11 | Felodipine | >1825 | -- |  | Atorvastatin Calcium | 29 | DA | 29 |
| PT2-12 | No medication |  | -- |  | Atorvastatin Calcium | 36 | DA | 36 |
| PT2-13 | Amlodipine Besylate；amlodipine；Irbesartan | >1825；14d；12d | -- |  | Atorvastatin Calcium | 15 | DA | 15 |
| PT2-14 | Missing |  | Missing |  | Missing |  |  |  |
| PT2-15 | Losartan potassium | >1825 | Metformin | 35 | -- |  | DA | 19 |
| PT2-16 | Felodipine | >1825 | -- |  | Atorvastatin Calcium | 21 | DA | 21 |
| PT2-17 | Nimodipine | >1825 | Metformin | >1825 | Atorvastatin Calcium | 21 | DA | 21 |
| PT2-18 | -- |  | -- |  | Atorvastatin Calcium | 24 | DA | 24 |
| PT2-19 | amlodipine | 11 | Gliclazide；Metformin；Acarbose | 11 | Atorvastatin Calcium | 18 | SA | 18 |
| PT2-20 | Compound Reserprine Tablets; Valsartan | 1460；1470 | -- |  | / |  | DA | 16 |
| PT2-21 | Missing |  | Missing |  | Missing |  | DA | 18 |
| PT2-22 | No medication |  | No medication |  | Atorvastatin Calcium | 15 | DA | 15 |
| PT2-23 | Felodipine | 5 | -- |  | Atorvastatin Calcium | 19 | DA | 19 |
| PT2-24 | amlodipine | 8 | -- |  | Atorvastatin Calcium | 23 | DA | 23 |
| PT2-25 | Missing |  | Missing |  | Missing |  | DA | 26 |
| PT2-26 | Missing |  | Missing |  | Missing |  | DA | 15 |
| PT2-27 | -- |  | -- |  | -- |  | DA | 15 |
| PT2-28 | amlodipine | 1 | -- |  | Atorvastatin Calcium | 19 | DA | 19 |

The units of numbers are days. DA, Dual Antiplatelet. SA, Single Antiplatelet**. --**, There is no disease. **/**, Sick and taking medication, but do not know the specific drug name. No medication, Sick but not taking medication. Missing, Related data loss.
